# Editorial: The neurogenetics of brain wiring

**DOI:** 10.3389/fgene.2025.1699487

**Published:** 2025-10-13

**Authors:** Ting Feng, Wenfeng Chen, Aja McDonagh, Zhenxing Liu

**Affiliations:** ^1^ Department of Biology, University of Nevada, Reno, NV, United States; ^2^ College of Biological Science and Engineering, Fuzhou University, Fuzhou, Fujian, China; ^3^ State Key Laboratory of Resource Insects, Institute of Apicultural Research, Chinese Academy of Agricultural Sciences, Beijing, China

**Keywords:** neurogenetics, neural network, neurological diseases, Huntington’s disease (HD), neurodegeneration

Understanding how the neural networks and circuits are guided by genetic programing is one of the most compelling frontiers in neuroscience. A wide array of neurological and psychiatric conditions, including age-related frailty, Huntington’s disease, leukodystrophies and gliomas, are increasingly recognized as disorders of neural circuitry rather than isolated cell-autonomous defects. This Research Topic, “*The Neurogenetics of Brain Wiring”*, aimed to explore how genetic variation shapes brain architecture and wiring, how these pathways interact with environmental cues, and how dysregulation of these processes leads to disease in nerve system. This editorial summarized key findings from the articles included in this topic, providing new insights into genetic mechanisms that sculpt neural circuits, through different experimental approaches and distinct perspectives.

The topic began with the article by Tao et al. which revealed that specific immune-related genes and immune cells within the tumor microenvironment (TME) are crucial for predicting patient outcomes. This research highlights a critical aspect of neurogenetics that goes beyond the wiring of neurons themselves: the genetic factors influencing the cellular and immune microenvironment of the brain. The article shows that understanding the genetic signatures of the TME can provide new prognostic indicators and potential therapeutic targets for neurological diseases, including cancer. Similarly, Passchier et al. updated identified variants and the physiological basis of the disease, and elaborated the gene variants that lead to the characteristic white matter edema, offering critical insights into how single-gene mutations can profoundly disrupt the myelin stability, which disrupts nerve signal transmission. This work not only serves as a valuable resource for clinicians and researchers but also indicates the direct link between specific genetic abnormalities and the structural integrity of the brain.

Frailty is a geriatric syndrome characterized by decreased physiological reserve and increased vulnerability to stressors. While frailty can be assessed by phenotypic criteria, understanding its genetic basis can aid in diagnosis and potentially reduce the adverse outcomes. By employing a cross-tissue transcriptome-wide association study (TWAS), Lin et al. identified two novel genes, *LRPPRC* and *HTT*, influencing frailty. Strikingly, the analysis further identified enrichment in immune and inflammatory signaling, cognition and second messenger production, linking frailty to both neural and immune pathways. These findings illustrate how genetics can unravel the complex genetic architecture of conditions that affect both the nervous system and overall physiological function. This work bridges the gap between aging research and neurogenetics, underscoring that the principles of brain wiring extend to its maintenance and eventual decline over a lifespan.

In a different but equally compelling line of inquiry, Pinto et al. addresses a critical methodological challenge in neurogenetic research. By re-evaluating Brodmann Area 9 (BA9) (prefrontal cortex) samples using propensity score matching to select controls age-matched to Huntington’s disease (HD) gene carriers, the authors investigate how the age of control groups can introduce confounding variables in studies aiming to identify differentially expressed genes (DEGs) in neurodegenerative diseases like HD. They demonstrate that using older control individuals can lead to the misidentification of DEGs, as age-related transcriptional changes can be mistaken for disease-specific effects. This article provides an important cautionary tale for the field, emphasizing the need for rigorous experimental design and age-matched controls. The findings have broad implications for transcriptomic studies across all neurogenetic disorders, as they highlight a potential pitfall that could lead to false conclusions and hinder the discovery of effective therapeutic targets.

Despite their advances, these studies share some limitations. Most of these studies rely heavily on publicly published genomic and transcriptomic datasets, such as GWAS (genome-wide association studies) cohorts, CGGA (Chinese Glioma Genome Atlas) and TCGA (the Cancer Genome Atlas), which are invaluable but are predominantly composed of participants of European or East Asian ancestry. This raises concerns about generalizability of their findings to more diverse global populations. Transcriptomic associations, while powerful, remain correlational and demand functional validation in cellular or animal models. Sample sizes, especially in the Huntington’s disease reanalysis and the MLC variant survey, constrain the strength of conclusions. Even in large-scale glioma analyses, predictive models require prospective validation before clinical translation. Moreover, across all four contributions, the gap between computational or descriptive findings and actionable therapies remains wide. However, the articles in this Research Topic offer a powerful testament to the diversity and depth of neurogenetic research. The future of neurogenetics lies in our ability to build on these foundations to deepen integration of multi-omics with functional neuroscience, and move findings from statistical associations to mechanistic and therapeutic insight. The goal is not just to understand the genetic blueprint of the brain but to use this knowledge to develop targeted therapies that can correct developmental errors, halt neurodegeneration, and restore healthy brain function.

